# A meta-analysis and The Cancer Genome Atlas data of prostate cancer risk and prognosis using epithelial cell adhesion molecule (EpCAM) expression

**DOI:** 10.1186/s12894-019-0499-8

**Published:** 2019-07-19

**Authors:** Yu Hu, Qiong Wu, Jialin Gao, Yongrui Zhang, Yuantao Wang

**Affiliations:** 1 Department of Pathology, China-Japan Union Hospital, Jilin University, Changchun, Jilin China; 2 Department of Urology, The First Hospital of Jilin University, No. 71 Xinmin Street, Changchun, 130021 Jilin China

**Keywords:** EpCAM, Expression, Bone metastasis, Prognosis, Prostate cancer

## Abstract

**Background:**

Epithelial cell adhesion molecule (EpCAM) expression has been reported in many types of cancer, including prostate cancer (PCa). However, the role of EpCAM expression remains inconsistent. We conducted a meta-analysis to assess the clinicopathological and prognostic significance of EpCAM expression in PCa.

**Methods:**

Publications were searched online using electronic databases. The available data were obtained from The Cancer Genome Atlas (TCGA). The odds ratios (ORs) or hazard ratios (HRs) with their 95% confidence intervals (CIs) were calculated.

**Results:**

We identified seven studies in which immunohistochemistry was used and that included 871 prostatic tissue samples. EpCAM expression was significantly higher in PCa samples than in benign and normal tissue samples (OR = 77.93, *P* = 0.002; OR = 161.61, *P* <  0.001; respectively). No correlation of EpCAM overexpression with pT stage and lymph node metastasis was observed; however, EpCAM overexpression showed a significant correlation with Gleason score (OR = 0.48, *P* = 0.012) and bone metastasis (OR = 145.80, *P* <  0.001). Furthermore, TCGA data showed that EpCAM overexpression was not closely correlated with age, pT stage, lymph node metastasis, number of lymph node, prostate-specific antigen level, Gleason score, biochemical recurrence, and overall survival. Based on multivariate Cox proportional-hazards regression analysis, a significant correlation was observed between EpCAM overexpression and 5-year worse biochemical recurrence free-survival.

**Conclusions:**

EpCAM overexpression may be correlated with the development of bone metastasis and worse biochemical recurrence free-survival of PCa. Further studies are needed to verify these findings.

## Background

Prostate cancer (PCa) is the second commonest malignancy and the fifth leading cause of cancer-related deaths in men [1]. Based on GLOBOCAN estimates, approximately 1.3 million new cases were clinically diagnosed with PCa in 2018, leading to approximately 359,000 PCa-related deaths worldwide [1]. Several therapeutic strategies, including radical prostatectomy and radiotherapy, have shown a better clinical outcome for patients with early-stage PCa [2, 3]. In contrast, patients with advanced stage PCa have distant metastases and consequently, worse prognosis because of the lack of the effective treatment options [4, 5]. Therefore, a novel molecular biomarker is required to improve the prognosis of patients with PCa.

Cancer stem cells (CSCs) are a small group of cells within tumors and are responsible for self-renewal, uncontrolled differentiation, and tumorigenicity [6, 7]. CSCs contribute to cancer development, progression, and metastasis [8–10]. Epithelial cell adhesion molecule (EpCAM), known as epithelial-specific antigen (ESA) or CD326, a membrane glycoprotein, plays an important role in Ca2+ independent hemophilic cell-to-cell adhesion, cell signaling, migration, proliferation, and differentiation [11, 12]. The presence of CSCs in PCa may partially play a role in cancer progression, metastasis, and chemoresistance [13, 14]. EpCAM is identified as a CSC marker and a potential therapeutic target for cancer [15]. EpCAM is expressed in many types of human cancer, such as breast cancer, gastric cancer, and colorectal cancer [16–18]. Recent studies also demonstrated that high EpCAM expression may predict poor clinical outcome in breast cancer [19], ovarian carcinoma [20], and hepatocellular carcinoma [21]. Some studies reported that EpCAM was frequently expressed and associated with worse prognosis of patients with PCa [22, 23].

The development of PCa develops involves the transition of normal epithelium to benign prostatic epithelium, and subsequent progression to malignant carcinoma through multiple sequences [24–26]. The role of EpCAM expression in PCa development and progression remains controversial. Ni 2013 et al. reported that the frequency of EpCAM expression was similar in PCa and benign prostatic tissue samples [22]. In contrast, Li et al. showed that EpCAM expression was notably higher in PCa than benign prostatic tissue samples [27]. Thus, the primary objective of this study was to identify the role of EpCAM in determining the risk of PCa development. The secondary objective was to perform a meta-analysis to assess the clinicopathological and prognostic value of EpCAM in PCa.

## Methods

### Search strategy from meta-analysis

This meta-analysis was conducted based on the preferred reporting items for systematic reviews and meta-analyses (PRISMA) statement criteria [28]. Articles published until December 1, 2018 were systematically searched in PubMed, EMBASE, Cochrane Library, EBSCO, Wanfang, and CNKI databases. The following keywords and search terms were used: “epithelial cell adhesion molecule OR EpCAM OR Ep-CAM OR CD326 OR 17-1A antigen OR GA733 OR CO17-1A OR EGP OR KS1-4 OR ESA OR MOC31 OR BerEP4 OR TACSTD1 OR TROP1,” “prostate OR prostatic,” “cancer OR carcinoma OR tumor OR neoplasm.” We also manually screened the reference lists of the eligible studies to find other potential publications.

### Selection criteria from meta-analysis

Studies that met the following criteria were selected: 1) patients were diagnosed with PCa by histopathological examination; 2) the immunohistochemical assessment of EpCAM using anti-EpCAM antibody was defined as a positive based on the publications; 3) studies provided the available data for the association of EpCAM expression between PCa and nonmalignant control groups; 3) studies provided the available data for the correlation of EpCAM expression with the clinicopathological features of patients with PCa; 4) studies provided sufficient data to evaluate the prognostic role of EpCAM expression using multivariate Cox proportional-hazards regression analysis. Only the latest or more accurate study was included when authors published several papers using duplicated sample data. The exclusion criteria were as follows: 1) letters, case reports, editorials, and studies conducted in animals and cell lines; 2) studies that lacked sufficient data.

### Data extraction from meta-analysis

Data were extracted from all eligible studies independently by two authors. Discrepancies were resolved by a third author. The following information was extracted: name of the first author’s names, year of publication, country, ethnicity and age of the patients, tumor stage, detection method, antibody, cut-off values, expression frequency, number of cancer patients and nonmalignant controls, clinicopathological characteristics, and survival data.

### TCGA dataset

The RNA-sequencing PCa data and corresponding clinical information were downloaded from TCGA (https://cancergenome.nih.gov/). Finally, 495 PCa patients with the available clinical information and 52 normal prostatic samples were included.

### Statistical analysis

Data were obtained from the original articles. The corresponding author of each study with an available email was also contacted to request useful information on missing or incomplete data. The pooled odds ratios (ORs) and the corresponding 95% confidence intervals (CIs) were used to estimate the difference in EpCAM expression between PCa and nonmalignant controls. The association of EpCAM expression with the clinicopathological parameters of PCa were also calculated using pooled ORs with 95% CIs. Based on multivariate Cox proportional-hazards regression analysis, the overall hazard ratios (HRs) with 95% CIs were used to determine the prognostic impact of EpCAM expression. The statistical heterogeneity between studies was estimated using the Cochran’s Q statistic [29]. The random-effects model (DerSimonian and Laird method) was used to improve the reliability of the data [30]. When significant heterogeneity was present (*P* < 0.1), a sensitivity analysis was performed to evaluate the influence and stability of one study on the results by deleting a single study [31]. Publication bias was measured using Egger’s linear regression test (≥ 10 studies) [32]. Meta-analysis was performed using Stata software (version 12.0, Stata Corporation, College Station, TX, US).

For TCGA dataset, the difference in EpCAM expression between PCa and normal prostatic tissues was determined using the t-test. The relationship between EpCAM expression and the clinical features was analyzed using the univariate logistic regression model. Survival curve were analyzed by Kaplan-Meier method and log-rank test. The univariate and multivariate Cox proportional-hazards regression analyses were used to determine the influence of EpCAM expression on survival. The patients were divided into high and low expression groups based on the median value of EpCAM expression. TCGA data were conducted using R version 3.5.1 (R Core Team, 2018).

## Results

### Characteristics of the eligible studies from meta-analysis

**Fig. 1:**
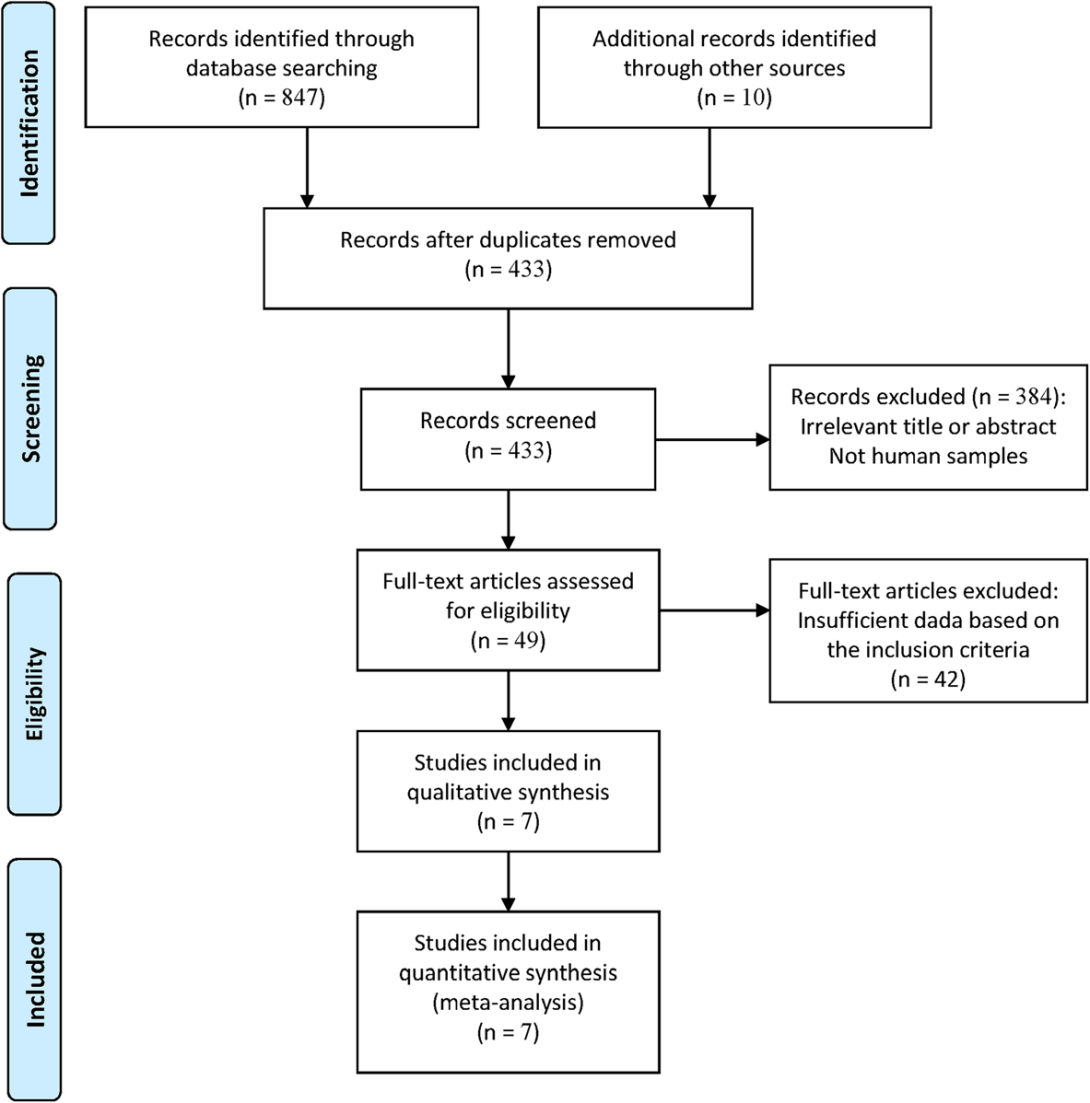
Flow diagram of the study selection

Figure 1 shows the detailed procedure of the selecting the publications. Based on the selection criteria, seven studies were finally selected; in these studies, immunohistochemistry (IHC) was performed to determine EpCAM expression in patients with PCa [22, 23, 27, 33–36], (PCa tissue samples, *n* = 671; normal prostatic tissue samples, *n* = 30; benign prostatic tissue samples, *n* = 170). Four studies involving 536 patients with PCa reported data related to EpCAM expression and clinicopathological characteristics [27, 33, 34, 36]. One study reported the prognostic information using multivariate Cox proportional-hazards regression analysis in patients with PCa. The basic characteristics of the included publications are listed in Table 1.

**Table 1:** The basic characteristics of the eligible studies

First author	Country	Age	Stage	Antibodies	Positivity (IHC)	Control groups	Cancer	Control	GS (≥ 7)	GS (≤ 6)	T stage 3–4	T stage 1–2	Lymph node metastasis (Yes)	Lymph node metastasis (No)	Bone metastasis (Yes)	Bone metastasis (No)	Clinical outcome-MA
							N (%)	N (%)	E+/N	E+/N	E+/N	E+/N	E+/N	E+/N	E+/N	E+/N	
Went 2006 [36]	Switzerland	NA	T1-T4	ESA, clone VU-1D9, Novocastra, Newcastle upon Tyne, UK, dilution 1: 50	> 70%		414 (87.2%)		138/168	223/246	82/89	276/321	8/10	329/378			No
Mukherjee 2009 [35]	USA	NA	NA	Abcam, cat. #11294, dilution 1:200	≥ 20%	Normal	23 (91.3%)	20 (0%)									No
Benko 2013 [23]	Croatia	65	T2-T3	ab32392, ABCAM, Cambridge, MA, dilution 1:600	≥ 40%	Benign	102 (52%)	102 (1%)									Yes
Ni 2013 [22]	Australia	NA	NA	Epitomics, Inc.dilution 1:100	≥ 25%	Benign	10 (100%)	10 (90%)									No
Ni 2013 [22]	Australia	NA	NA	Epitomics, Inc.dilution 1:100	≥ 25%	Normal	10 (100%)	10 (20%)									No
Rybalov 2014 [34]	The Netherlands	NA	T2-T4	Clone VU-1D9, Leica Biosystems, Newcastle, UK, dilution 1:100	Weak-strong		17 (82.4%)		11/14	2/2	11/14	3/3					No
Li 2015 [27]	China	NA	NA	NA	1–9 scores	Benign	63 (88.9%)	58 (0%)							20/20	2/5	No
Campos 2016 [32]	Mexico	NA	NA	clone VU-1D9, Leica Biosystems, Newcastle, UK, dilution 1:100	TIS ≥ 1		42 (50%)						21/21	0/21	19/20	0/14	No

*N* number of the study population, E+ positive expression, *MA* multivariate analysis, *NA* not applicable, *TIS* total immunostaining score, *IHC* immunohistochemistry

### Association between EpCAM expression and PCa risk from meta-analysis

**Fig. 2:**
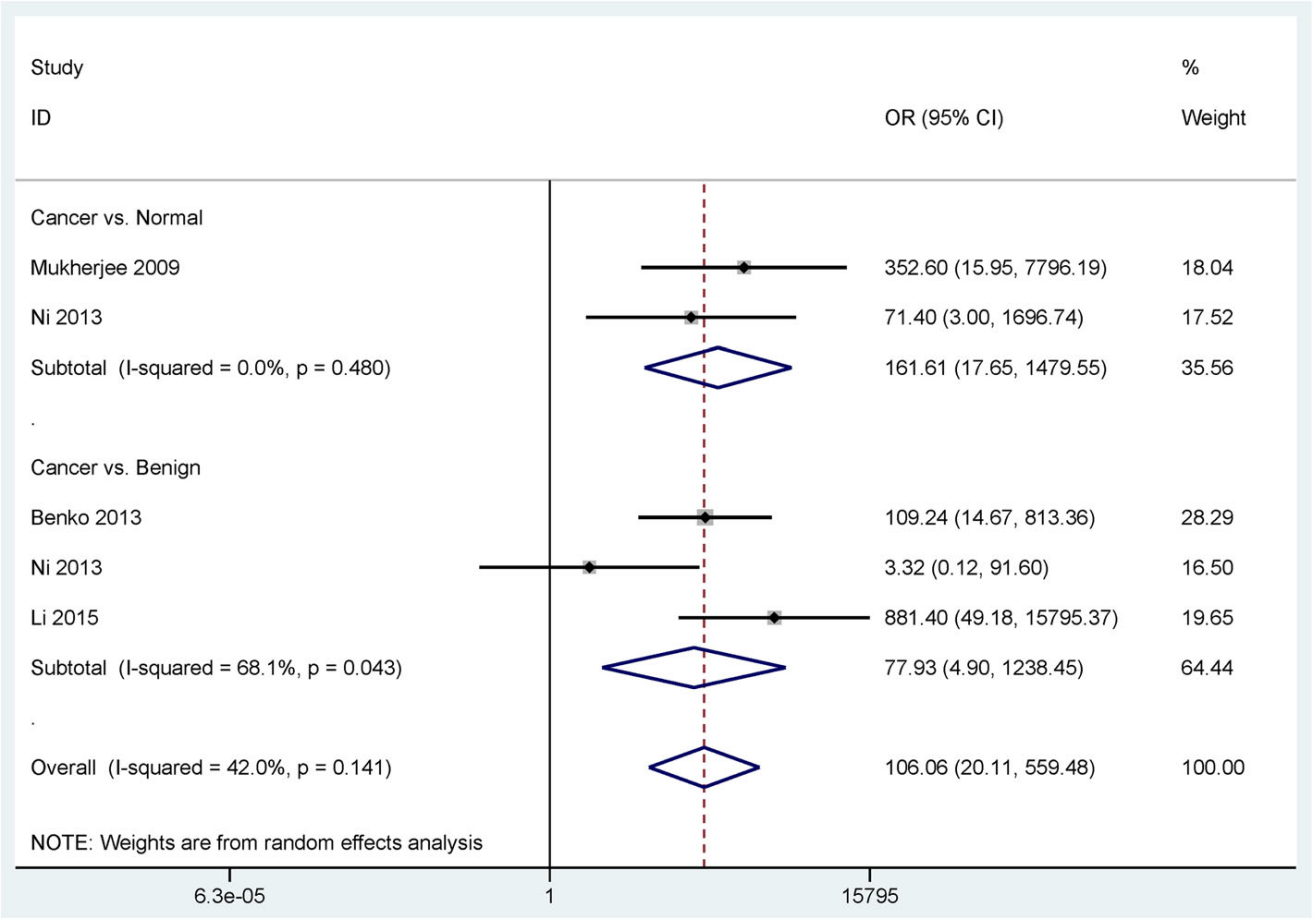
Forest plot of the association between EpCAM immunoexpression and prostate cancer

175 PCa tissue samples were compared with 170 benign prostatic tissue samples and 33 PCa tissue samples were compared with 30 normal prostatic tissue samples (Table 2). The results showed that EpCAM expression in PCa was significantly higher than in benign and normal tissue samples (OR = 77.93, 95% CI = 4.90–1238.45, *P* = 0.002; OR = 161.61, 95% CI = 17.65–1479.55, *P* < 0.001; respectively; Fig. 2).

**Table 2:** Summary of the pooled results from meta-analysis

Group	Studies	Cancer samples	Nonmalignant samples	OR with 95% CI	*P*	SE
Cancer vs. Benign	3	175	170	77.93 (4.90–1238.45)	0.002	1.411
Cancer vs. Normal	2	33	30	161.61 (17.65–1479.55)	< 0.001	1.13
Clinicopathological characteristics						
Gleason score (≥ 7 vs. ≤ 6)	2	430		0.48 (0.27–0.85)	0.012	0.293
T stage (T3–4 vs. T1–2)	2	427		1.75 (0.78–3.91)	0.175	0.411
Lymph node metastasis (Positive vs. Negative)	2	430		27.89 (0.004–1.7e+ 05)	0.455	4.481
Bone metastasis (Positive vs. Negative)	2	59		145.80 (14.58–1458.02)	< 0.001	1.175

*OR* odds ratio, *95% CI* 95% confidence interval, *SE* standard error

Significant heterogeneity was detected between PCa and benign prostatic tissue samples (*P* = 0.043 < 0.1). To evaluate the change in pooled OR and heterogeneity based on the omission of an individual study, a sensitivity analysis was performed (PCa versus benign controls). When the study of Ni 2013 et al. [22] was removed, the re-calculated OR was 237.96 (95% CI = 32.59–1737.73, *P* < 0.001), resulting in a significantly decreased heterogeneity (*P* = 0.242).

### Association between EpCAM expression and clinicopathological characteristics from meta-analysis

**Fig. 3:**
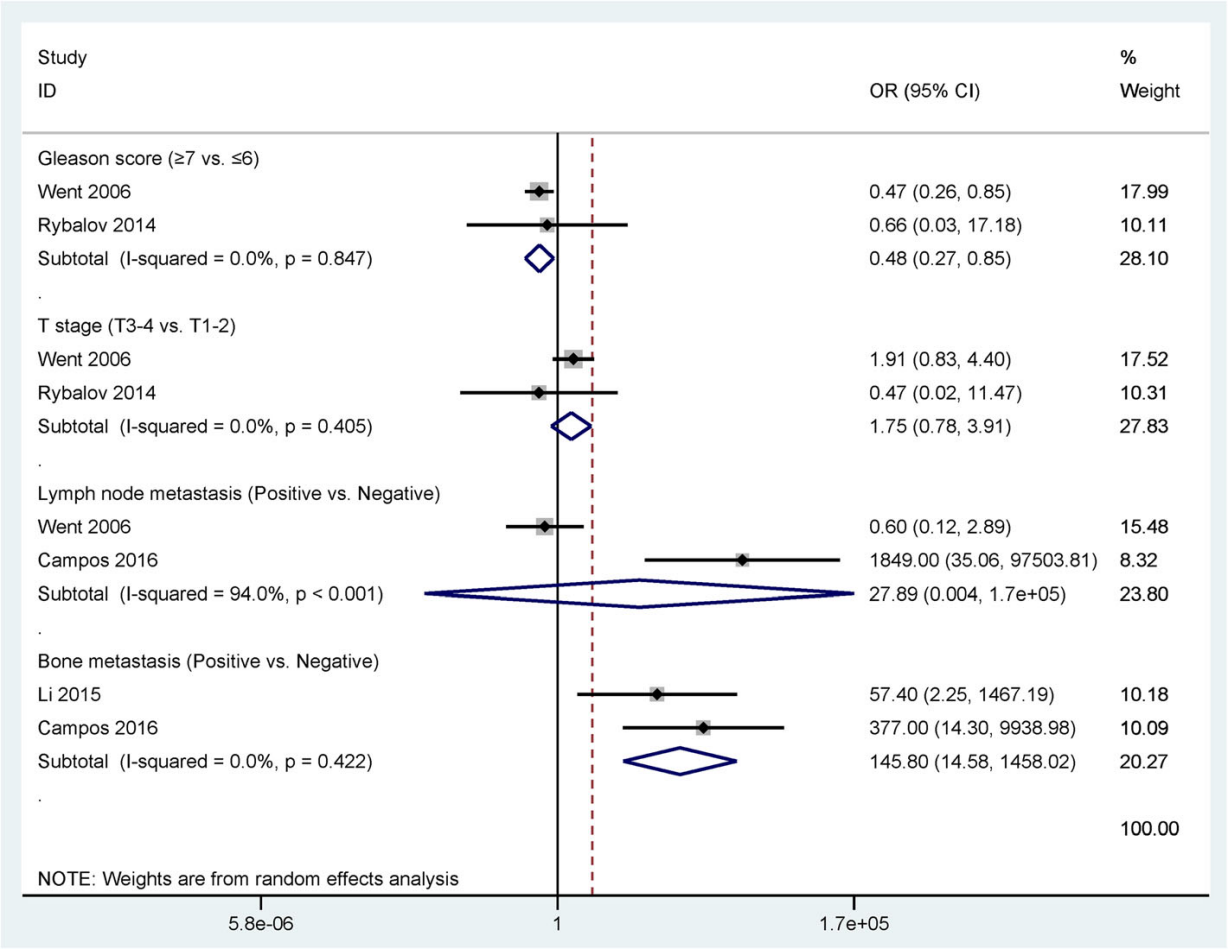
Forest plot of the association of EpCAM immunoexpression with the clinicopathological features of patients with prostate cancer

Data from two studies with 427 patients with PCa showed that EpCAM overexpression was not linked to pT stage (*P* = 0.175). Data from two studies with 430 PCa cases demonstrated no relationship between EpCAM overexpression and lymph node metastasis (*P* = 0.455; Fig. 3).

Data from two studies with 430 PCa cases demonstrated that EpCAM overexpression was associated with Gleason score (≥ 7 vs. ≤ 6 scores: OR = 0.48, 95% CI = 0.27–0.85, *P* = 0.012; Fig. 3). Data from two studies with 59 patients with PCa showed that EpCAM overexpression was closely correlated to bone metastasis (OR = 145.80, 95% CI = 14.58–1458.02, *P* < 0.001; Fig. 3). Moreover, no heterogeneity was measured between EpCAM overexpression and the Gleason score and bone metastasis of PCa (*P* > 0.1).

### Association between EpCAM expression and PCa prognosis from meta-analysis

A study reported that EpCAM overexpression was significantly correlated with 5-year worse biochemical recurrence free-survival using multivariate Cox proportional-hazards regression analysis (HR = 6.79, 95% CI = 2.38–19.45) in 102 patients with PCa [23].

### TCGA

Four hundred ninety-five PCa cases with the available clinical information and 52 normal prostatic tissue samples were identified from TCGA. EpCAM expression level was notably higher in PCa than in normal prostatic tissues (*P* < 0.001; data not shown).

Univariate analysis using logistic regression showed that EpCAM overexpression was not significantly associated with the clinicopathological characteristics of PCa (Table 3), including age (*P* = 0.065), pT stage (T3–4 vs. T1–2: *P* = 0.78), lymph node metastasis (positive vs. negative: *P* = 0.889), number of lymph node (> 10 vs. ≤ 10: *P* = 0.39), prostate-specific antigen (PSA) value (  ≥ 10 vs. < 10: *P* = 0.288), Gleason score (≥  7 vs. ≤  6: *P* = 0.561), and biochemical recurrence (yes vs. no: *P* = 0.408).

**Table 3:** Association of EpCAM overexpression with the clinicopathological characteristics from The Cancer Genome Atlas (TCGA)

Factors	Total (N)	OR with 95% CI	*P*
Age (≥ 61 vs. < 61)	495	0.72 (0.5–1.02)	0.065
T stage (T3–4 vs. T1–2)	488	0.95 (0.66–1.37)	0.78
Lymph node metastasis (Positive vs. Negative)	422	1.04 (0.63–1.69)	0.889
Number of lymph node (> 10 vs. ≤ 10)	420	1.18 (0.81–1.74)	0.39
Biochemical recurrence (Yes vs. No)	427	0.79 (0.45–1.38)	0.408
Gleason score (≥ 7 vs. ≤ 6)	483	1.19 (0.66–2.14)	0.561
PSA value ( ≥ 10 vs. < 10)	438	1.75 (0.62–4.89)	0.288

*N* number of the study population, *PSA* prostate-specific antigen, *OR* odds ratio, *95% CI* 95% confidence interval

To further evaluate the prognostic value of EpCAM overexpression in patients with PCa, Kaplan-Meier survival analysis was performed. This revealed that EpCAM overexpression was not correlated with the prognosis of overall survival (*P* = 0.25; data not shown).

## Discussion

CSCs, a special subpopulation of tumor cells, have the ability to form the bulk of the tumor and contribute to tumor progression and relapse [9]. Therefore, eradication of CSCs is considered as a crucial challenge for a successful cancer therapy [37]. EpCAM is identified as a CSC marker that is closely linked to tumor progression [38]. EpCAM plays an important role in the cell-cell adhesion, cell signaling, migration, proliferation, and differentiation [39]. EpCAM also plays an important role in mediating migration of immune cells [38]. EpCAM in PCa is associated with tumor progression and metastasis and therapeutic resistance via the PI3K/Akt/mTOR signaling pathway [22]. EpCAM is frequently overexpressed across a wide range of human cancer, including pancreatic adenocarcinoma, breast cancer, ovarian cancer, and head and neck squamous cell cancer [40]. EpCAM overexpression is correlated with worse prognosis in some types of cancer [19, 41, 42]. Some studies reported that EpCAM is highly expressed in patients with PCa [22, 27, 33, 34]. However, the function of EpCAM and its clinical effect in PCa remains largely unclear. In this study, we analyzed the clinicopathological significance of EpCAM expression and its role in the prognosis of PCa.

The pooled data demonstrated that EpCAM expression was significantly higher in PCa than in benign and normal prostatic tissue samples, suggesting that EpCAM overexpression may be associated with the development of PCa. Our results were consistent with the previous studies that EpCAM was expressed more frequently in PCa than in benign [23, 27] and normal prostatic tissue samples [22, 35]. Moreover, TCGA data also showed that EpCAM expression was notably increased in PCa than in normal tissue samples.

We further analyzed whether EpCAM overexpression was correlated with the clinicopathological characteristics of patients with PCa. EpCAM overexpression was not linked to pT stage and lymph node metastasis, but was associated with Gleason score and bone metastasis. TCGA data revealed that EpCAM overexpression was not closely correlated with the clinicopathological characteristics in 495 patients with PCa, including age, pT stage, lymph node metastasis, number of lymph node, prostate-specific antigen (PSA) level, Gleason score, and biochemical recurrence. The pooled analyses suggested that EpCAM overexpression may predict bone metastasis. However, as the sample size was small, the relationship between EpCAM overexpression and bone metastasis requires further investigation.

EpCAM overexpression was reported to be closely associated with 5-year worse biochemical recurrence free-survival using multivariate Cox proportional-hazards regression analysis in 102 patients with PCa [23]; this suggests that EpCAM overexpression may be a potential prognostic biomarker for predicting poor biochemical recurrence free-survival. However, no association was found between EpCAM overexpression and overall survival of patients with PCa.

Our result showed no correlation between EpCAM overexpression and lymph node metastasis among two studies with 430 patients with PCa, which was consistent with the study of Went 2006 et al. [36] among a large cohort (388 patients). Heterogeneity was found in the correlation of PCa and benign prostatic tissue samples (*P* = 0.043). When we removed the study by Ni 2013 et al. [22], the re-calculated OR remained significant with no obvious evidence of heterogeneity. The analysis suggested that our results were reliable. The reasons of the heterogeneity were not very clear; the inappropriate conditions of IHC methods may cause the bias.

There were some limitations in the current study. First, as none of the present meta-analyses included ≥10 studies, we could not examine and rule out the risk of the potential publication bias. Although we systematically searched PubMed, EMBASE, Cochrane Library, EBSCO, Wanfang, and CNKI databases to minimize the potential bias, the eligible studies were restricted to papers published in English or Chinese. Moreover, studies published in language other than English and Chinese, unpublished papers, or conference abstracts were excluded based on incomplete information. Furthermore, studies with negative results are less likely to be published than papers with positive results. These reasons may cause potential bias. Second, the main ethnic population in this meta-analysis was European; other ethnic groups, such as Asians and Africans, were under-represented. Third, the studies regarding the associations of EpCAM overexpression with bone metastasis and biochemical recurrence free-survival were relatively small. Further studies with large sample size are needed to further confirm these results. Finally, the cut-off values of EpCAM expression were different among some studies, which showed inconsistent definitions for “negative” and “positive,” leading to the potential heterogeneity. Therefore, the results need to be interpreted with caution. EpCAM expression should be defined as positive or negative based on a uniform standard in the future.

## Conclusions

In conclusion, the current meta-analysis suggested that EpCAM expression was significantly high in PCa than in benign and normal prostatic tissue samples. EpCAM overexpression was not associated with age, pT stage, lymph node metastasis, number of lymph node, prostate-specific antigen (PSA) level, biochemical recurrence, and overall survival. EpCAM overexpression may be related to the development of bone metastasis and worse biochemical recurrence free-survival of PCa. Our results should be interpreted with caution owing to the small sample size. Additional large-scale prospective studies are required to further validate our findings.

## Data Availability

The datasets used and/or analyzed during the current study are available from the corresponding author on reasonable request.

## Abbreviations

CIs: Confidence intervals
CSCs: Cancer stem cells
EpCAM: Epithelial cell adhesion molecule
ESA: Epithelial-specific antigen
HRs: hazard ratios
IHC: Immunohistochemistry
ORs: The odds ratios
PCa: Prostate cancer
PSA: Prostate-specific antigen
TCGA: The Cancer Genome Atlas
